# Measurement precision of the Pain Catastrophizing Scale and its short forms in chronic low back pain

**DOI:** 10.1038/s41598-022-15522-x

**Published:** 2022-07-14

**Authors:** Franco Franchignoni, Andrea Giordano, Giorgio Ferriero, Marco Monticone

**Affiliations:** 1 Physical and Rehabilitation Medicine Unit, Scientific Institute of Tradate, Istituti Clinici Scientifici Maugeri, IRCCS, Via Roncaccio 16, Tradate, VA Italy; 2 Bioengineering Unit, Scientific Institute of Veruno, Istituti Clinici Scientifici Maugeri, IRCCS, Veruno, NO Italy; 3 Department of Biotechnology and Life Sciences, University of Insubria, Varese, Italy; 4 Department of Medical Sciences and Public Health, University of Cagliari, Cagliari, Italy; 5 Neurorehabilitation Unit, Department of Neuroscience and Rehabilitation, ARNAS G. Brotzu Hospital, Cagliari, Italy

**Keywords:** Diseases, Health care, Medical research, Signs and symptoms

## Abstract

The Pain Catastrophizing Scale (PCS) is a widely studied tool to assess pain catastrophizing for chronic low back pain (LBP). Short forms of the PCS exist, but their measurement precision at individual level is unclear. This study aimed to analyze the Rasch psychometric characteristics of the PCS and three of its short forms (two 4-item and one 6-item) in a sample of 180 Italian-speaking patients with chronic LBP, and compare their measurement precision at the individual level. We performed a Rasch analysis on each version of the PCS and calculated test information functions (TIFs) to examine conditional measurement precision. Rasch analysis showed appropriate rating category functioning, unidimensionality, and acceptable fit to the Rasch model for all PCS versions. This represented a prerequisite for performing further advanced psychometric analyses. According to TIFs, the PCS full scale showed—at any score level—higher measurement precision in estimating individual pain catastrophizing than its short forms (which had unacceptably high standard errors of measurement). Our results show acceptable conditional precision of the PCS full scale in estimating pain catastrophizing. However, further studies are needed to confirm its diagnostic accuracy at individual level. On the other hand, the study warns against use of the three PCS short forms for clinical decision-making at the individual level.

## Introduction

Non-specific low back pain (LBP) is a leading contributor to disease burden worldwide^1^, and the most common musculoskeletal disorder affecting the quality of life of individuals, especially if persistent^2^. Psychological factors may also contribute to the burden of these patients, in particular pain-related fear of movement (or kinesiophobia) and catastrophizing thoughts^3–5^.

Pain catastrophizing is defined as ‘*an exaggerated negative orientation towards actual or anticipated pain experiences*’^6^. It comprises a set of distorted and exaggerated emotional and cognitive reactions towards pain experiences leading to increased pain-related distress and disability.

The Pain Catastrophizing Scale (PCS) is a 13-item self-report questionnaire considered to be the most frequent and extensively studied tool to assess pain catastrophizing for chronic pain^6,7^. Good levels of content and construct validity, internal consistency and test–retest reliability of the PCS have been reported in studies examining different musculoskeletal disorders^8,9^ and different language versions^7^.

As for the scale dimensionality, some papers have described the PCS structure as tapping a single second-order latent construct (catastrophizing) characterized by three interrelated first-order factors (helplessness, rumination, and magnification)^6,10^, although a certain inconsistency in the item composition of the subdomains has been noted^7^. Other studies, applying Rasch analysis, have pointed out the substantial unidimensionality of the scale, despite the slightly different results found with some minor psychometric flaws^11–13^.

Recently, some shortened versions of the PCS have been developed, one with 6 items, PCS-6^14^, and two with 4 items, PCS-4^15^ and BriefPCS^16^. These short scales are often used to reduce assessment time and respondent and administrator burden when administered in clinical settings and for analyses at group level. However, although the shortened versions show a quite good total-score reliability, the measurement precision at individual level (i.e. the ability to provide accurate estimates of an individual’s true score and change score) has never been assessed across the different PCS versions. This is a crucial parameter to consider when making clinical decisions in the individual^17–19^. It can be examined only with item-response theory methods^18^.

The aim of this study was to analyze—in an Italian-speaking sample of individuals with chronic non-specific LBP—the main Rasch psychometric characteristics of the PCS and three short versions (PCS-6, PCS-4, and BriefPCS), in order to compare their measurement precision at individual level, through test information function, and so provide evidence for their appropriate utilization in clinical settings. Based on the drawbacks of scale shortening^17–19^, we hypothesized that measurement precision would be lower in each of the three PCS short versions^14–16^ than in the original 13-item PCS^6^.

## Methods

### Subjects

We conducted a secondary analysis of data from an observational study, aiming at examining the validity and reliability of the PCS in individuals with chronic non-specific LBP^20^. Outpatients referred, between January and June 2010, to our rehabilitation hospital and three affiliated rehabilitation centers from surrounding hospitals and by general practitioners were assessed for eligibility by a rehabilitation specialist. The inclusion criteria were chronic non-specific LBP, age ≥ 18 years, and ability to read and speak Italian fluently; the exclusion criteria were acute and subacute non-specific LBP, specific causes of LBP, central or peripheral neurological signs, systemic illness or psychiatric deficits, and recent myocardial infarction, cerebrovascular events, or chronic lung or renal diseases. A research assistant recorded patients’ demographic and clinical characteristics, which can be found in^20^. Subjects satisfying the inclusion criteria were informed about the research aims and procedures. Informed consent was obtained from all subjects and/or their legal guardian(s). No patient refused to participate in the questionnaire completion.

The Institutional Review Board of Salvatore Maugeri Foundation’s Scientific Institute of Lissone (Italy) approved the study (no. 08/09; date of approval: 12/02/2009). The study was conducted in conformity with ethical and human principles of research^20^.

### Outcome measures

#### Pain Catastrophizing Scale and its short forms

The PCS is a 13-item self-report questionnaire^6^. Patients are asked to rate the degree to which they have any of the thoughts described in the questionnaire using a 5-point Likert scale ranging from 0 (never) to 4 (always). The total score is the sum of the scores for the individual items, and ranges from 0 to 52. Higher scores reflect higher levels of catastrophic thoughts. The Italian cross-culturally adapted and validated version was used, which has shown satisfactory psychometric properties^20^.

For each of the three validated PCS short-forms, we created a database extracting the relevant information from the original 13-item PCS:

- The 6-item PCS (PCS-6)^14^ is composed of items # 4, 5, 6, 10, 11, and 13, with a score range 0–24. The authors created this version based on factor analytic studies, selecting two items from each of the three PCS factors: helplessness, rumination, and magnification.
- The 4-item PCS (PCS-4)^15,21^ consists of items # 3, 6, 8, and 11, with a score range 0–16. This version was originally validated in individuals with upper limb diagnoses^15,21^, and later extended to other musculoskeletal pain^13^. Item selection was based on item variance, item intercorrelations and reliability analyses.
- The 4-item BriefPCS-chronic (BriefPCS)^16^ consists of items # 4, 9, 10, and 11, with a score range 0–16. This quite new 4-item version was developed through triangulation procedures using conceptual, factor analytic and Rasch methods.

In all PCS versions, higher scores indicate a higher level of catastrophizing.

### Statistical analysis

Raw global scores of the four scales were correlated using Spearman’s rank correlation coefficient.

Rasch analysis was performed separately for each scale with Winsteps^®^ software v. 3.68.2 (Winsteps.com, Beaverton, OR, USA), using a rating scale model. Guidelines for model selection and a log-likelihood ratio test were used to decide which model to adopt (rating scale vs. partial credit)^22^. An introduction to this modern statistical technique and related concepts can be found in dedicated textbooks^23^. Applying Rasch analysis to the data from our sample of 180 individuals, we expected a stable calibration of items within ± ½ logit with 99% confidence^24^. A diagnostic assessment of the rating categories was performed in order to investigate whether each response category was being used effectively and consistently, according to criteria suggested by Linacre^25^.

Each scale (PCS, PCS-6, PCS-4, and BriefPCS) was assessed with the following procedure.

We examined the internal construct validity of the scale checking the fit of each item to the latent trait as per the Rasch model (goodness-of-fit test). Chi-square fit statistics (expressed as infit and outfit mean-square statistics, MnSq) for each item were calculated, considering MnSq between 0.70 and 1.30 as an indicator of acceptable fit^26^*.* Reliability was evaluated –according to Rasch methods– in terms of both item and person reliability index, indicating the degree of replicability of the estimates across other samples (range 0–1; coefficients > 0.80 are considered as good, > 0.90 as excellent)^23^.

Principal component analysis of standardized residuals (PCAR) was performed in order to verify the following psychometric properties: i) unidimensionality of the scale, examining variance explained by the Rasch factor (the dominant latent trait under measurement) and unexplained variance of the first factor after the Rasch factor was extracted; and ii) local independence of items in each scale^23^. No residual association among item responses should be found once the Rasch factor has been conditioned out. As suggested by Christensen et al.^27^, we considered any residual correlation for 2 items > 0.20 above the average observed residual correlation as an indicator of potential local dependence. Locally dependent items were accommodated using a traditional testlet approach, i.e. incorporating them into testlets (super-items)^28^.

Furthermore, the test information function (TIF) was calculated, as the sum of all the item information functions^23,29^. At any latent trait level (θ), the test information is the reciprocal of the variance of the estimates around the real value^18^. Thus, the TIF shows the amount of information (degree of measurement precision) provided by the test in estimating θ over the whole range of pain catastrophizing, according to the formula: SE (θ) = 1/√I(θ), where SE is the standard error of the estimated θ, and I(θ) is the information at θ. We defined as acceptable precision SE ≤ 0.5, while the corresponding value of information and the (approximate) reliability would then be > 4 and > 0.75, respectively^29,30^. However, according to classical test theory a minimum reliability of 0.85 to 0.90 is recommended for individual judgments^31^.

At any level of individual pain catastrophizing, the SE can be used to calculate the 95% confidence interval (CI_95_) for the true score (estimated latent score ± 1.96 SE). Then, to directly compare measurement precision between two scales with a different number of items, one needs to take the scale length into account computing the ‘relative’ CI_95_, i.e. the ratio between the CI_95_ for a given test score and the maximum score range in the scale^19^. With scale shortening, CI_95_ becomes narrower, but the score range also decreases, usually at a faster rate^32^. Consequently, CI_95_ for the shortened scale may encompass a larger proportion of the score range than that for the original scale; thus, the total score provides a less precise estimate of the true score, and of the individual change assessment^17^. Finally, we provided a practical example of the effect of different measurement precisions, comparing the scale performance—in terms of relative CI_95_—at the cut-off scores for clinically relevant level of catastrophizing suggested by the manual (30 out of 52 points) for the PCS^33^, and recently proposed by Walton et al.^16^ (9 out of 16 points) for BriefPCS.

### Ethical approval and consent to participate

Obtained.

### Consent for publication

All authors consent to the publication of the manuscript in this journal.

## Results

The study involved 180 individuals with chronic non-specific LBP: 77 women (43%) and 103 men (57%) with a mean age of 44.1 ± 11.3 years (range 18–73). The median duration of LBP was 12 months (range 3–48). Socio-demographic and clinical details are reported in the original prospective, single-group, observational study^20^.

Average scores and standard deviation for each scale were as follows: PCS = 18.1 ± 10.1 (possible score range: 0–52); PCS-6 = 7.9 ± 5.2 (possible score range 0–16), PCS-4 = 7.6 ± 3.6 (possible score range 0–12); and BriefPCS = 4.9 ± 3.9 (possible score range 0–12).

The correlations (Spearman) between scales ranged from 0.90 (CI_95_ 0.86–0.92: PCS-4 vs. BriefPCS) to 0.96 (CI_95_: 0.95–0.97: PCS vs. PCS-6).

Rasch rating scale diagnostics showed an appropriate category functioning for all PCS versions.

In PCS, we absorbed the local dependence between items # 8 to 11 by creating a testlet incorporating these four items, representing the PCS sub-dimension “Rumination”. No other significant between-item residual correlation emerged at PCAR. After that, all items fit the Rasch model for measuring “pain catastrophizing” (Infit MnSq between 0.77 and 1.26, with all ZStd < 2) barring three exceptions (Table 1). In PCS, item # 7 was underfitting (Infit MnSq 1.67; Outfit MnSq 1.65), while items # 2 (Infit MnSq 0.67; Outfit MnSq 0.67) and # 6 (Infit MnSq 0.64; Outfit MnSq 0.68) were overfitting. In PCS-6, item # 13 (Infit MnSq 1.29; Outfit MnSq 1.32) showed a borderline fit. In BriefPCS, item # 4 (Infit MnSq 1.31; Outfit MnSq 1.33) was slightly misfitting and item # 10 was overfitting (Infit MnSq 0.67; Outfit MnSq 0.66).

**Table 1:** Item calibrations (Measure) and fit information—expressed as infit and outfit mean square statistics (MnSq)—for the PCS and its three short forms.

Item number		PCS			PCS-6			PCS-4			BriefPCS		
	Label	Measure^a^	Infit MnSq	Outfit MnSq	Measure	Infit MnSq	Outfit MnSq	Measure	Infit MnSq	Outfit MnSq	Measure	Infit MnSq	Outfit MnSq
1.	I worry all the time about whether the pain will end	− 1.16	1.08	1.26									
2.	I feel I can't go on	0.74	0.67	0.67									
3.	It's terrible and I think it's never going to get any better	0.82	0.82	0.73				2.06	1.01	0.87			
4.	It's awful and I feel that it overwhelms me	1.60	0.86	0.64	1.8	0.94	0.83				2.04	1.31	1.33
5.	I feel I can't stand it any more	0.89	0.81	0.73	0.92	0.89	0.76						
6.	I become afraid that the pain may get worse	− 1.20	0.64	0.68	− 1.68	0.79	0.82	− 0.46	0.9	0.92			
7.	I keep thinking of other painful experiences	0.12	1.67	1.65									
8.	I anxiously want the pain to go away	− 2.25	0.83 (testlet)	0.96 (testlet)				− 1.8	1.18	1.15			
9.	I can't seem to keep it out of my mind	0.08									− 0.36	0.85	0.94
10.	I keep thinking about how much it hurts	0.22			0.09	0.87	0.82				− 0.13	0.67	0.66
11.	I keep thinking about how badly I want the pain to stop	− 0.66			− 1.02	1.18	1.11	0.21	0.91	0.87	− 1.56	1.04	0.97
12.	There is nothing I can do to reduce the intensity of the pain	0.74	1.13	1.16									
13.	I wonder whether something serious may happen	0.07	1.27	1.28	− 0.1	1.29	1.32						

^a^Item calibration before testlet creation (items 8–11).

Table 2 reports—for each of the four PCS versions—the Rasch results for: unidimensionality analysis (PCAR); person and item reliability indices (together with Cronbach’s alpha); mean and range of person ability estimates; and number of extreme (maximum and minimum) scores.

**Table 2:** Main results of Rasch analysis of the four PCS versions, regarding: unidimensionality analysis (principal component analysis of the residuals); number of underfitting items; reliability indices (Rasch person and item reliability, and Cronbach’s alpha); mean and range of person ability estimates; and number of extreme (maximum and minimum) scores.

	PCS	PCS-6	PCS-4	BriefPCS
Variance explained by the Rasch factor	62.0%	68.8%	72.7%	73.7%
Eigenvalue of the first residual factor	1.9	1.8	1.8	1.5
Number of underfitting items	1	1	0	1
Person reliability	0.86	0.83	0.79	0.80
Item reliability	0.99	0.99	0.99	0.99
Cronbach’s alpha	0.90	0.87	0.80	0.86
Mean person ability (range), in logits	− 1.33 (− 4.15 to 2.48)	− 1.35 (− 4.51 to 3.16)	− 0.21 (− 4.15 to 4.40)	− 1.23 (− 4.55 to 4.54)
Minimum/maximum scores	3/0	6/0	4/0	30/0

**Figure 1:**
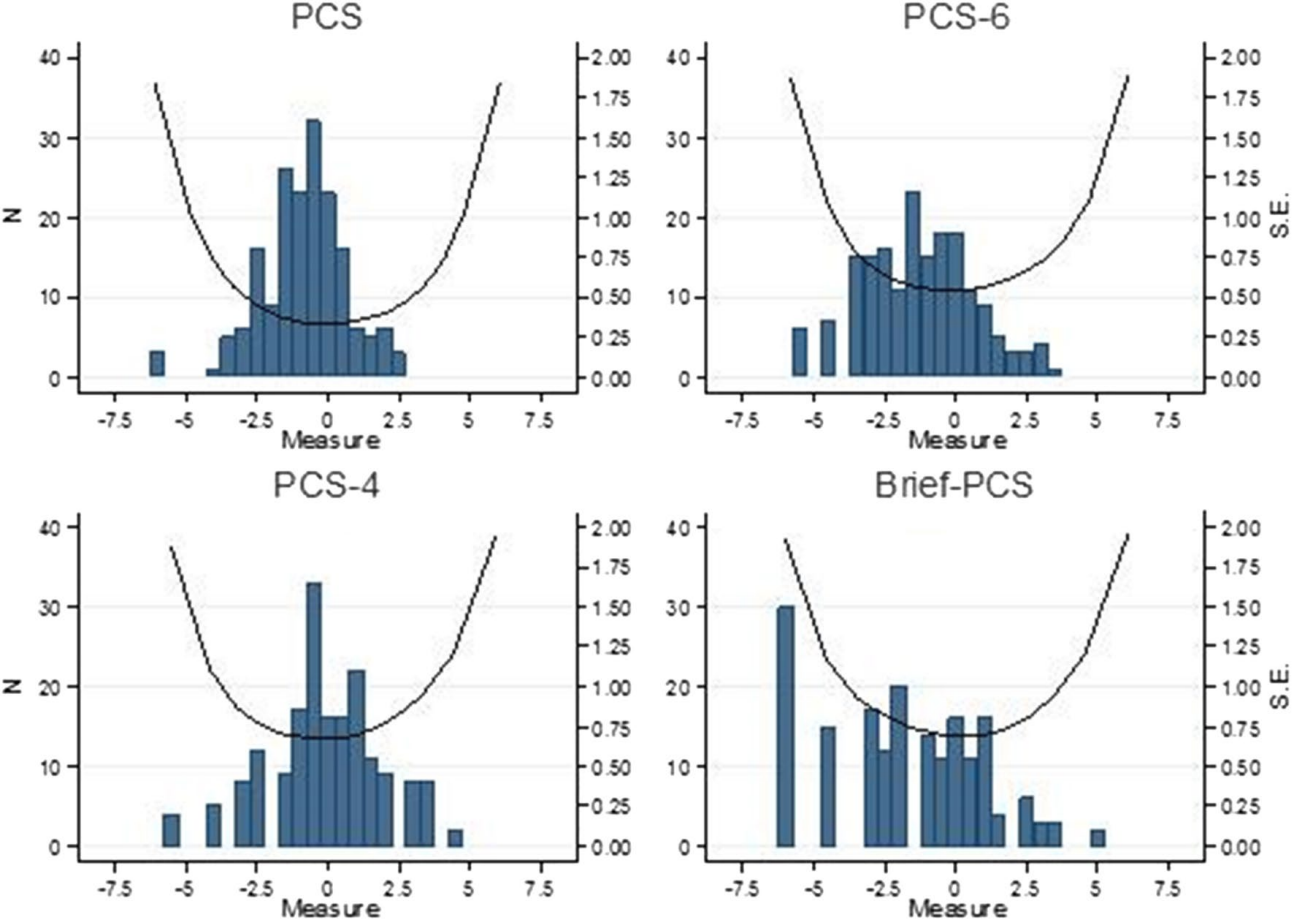
Standard Error of person ability estimates (SE, black line, right scale) and distribution of subject ability in our sample (measure, histograms, left scale), related to the four versions of the PCS: 13-item full version (PCS) and short forms (PCS-6, PCS-4, and Brief-PCS). An SE = 0.5 has been defined as the threshold for acceptable precision.

**Figure 2:**
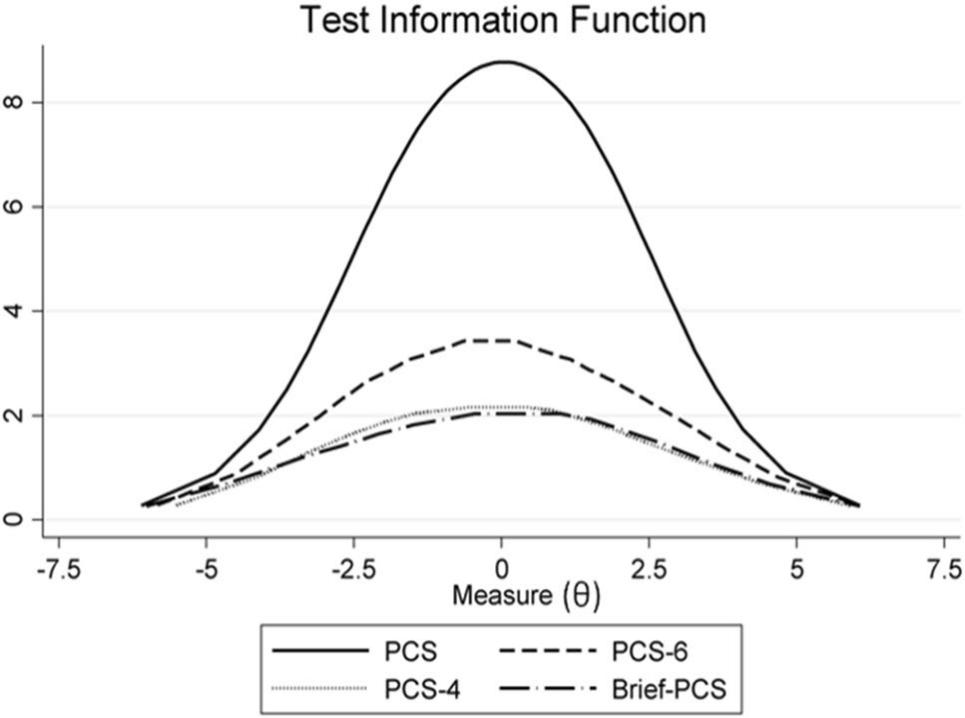
Test information function (TIF, in inverse-square-logits units) related to the four versions of the PCS: 13-item full version (PCS) and short forms (PCS-6, PCS-4, and Brief-PCS). The TIF is a graphical representation of the precision of each scale according to each individual pain catastrophizing level (θ). A TIF of 4 (roughly corresponding to a SE of 0.5) has been defined as the threshold for acceptable precision.

Figure 1 shows the SE of the four scales, as well as the range of subject ability as estimated by each scale, while Fig. 2 shows the TIFs. One can see that all scales showed their best precision in the same range of θ (about 0 logits), but:

- The PCS displayed a bell-shaped TIF, with an acceptable range of precision (SE ≤ 0.5) for person ability between about—3 and 3 logits;
- In PCS-6, PCS-4, and BriefPCS, the TIFs were always lower than 4 units (inverse-square-logits units), and measurement precision was low at any level of pain catastrophizing, with an SE always > 0.5 (higher in PCS-4 and BriefPCS than in PCS-6);
- The BriefPCS showed a subverted, right skewed distribution of subject ability.

**Figure 3:**
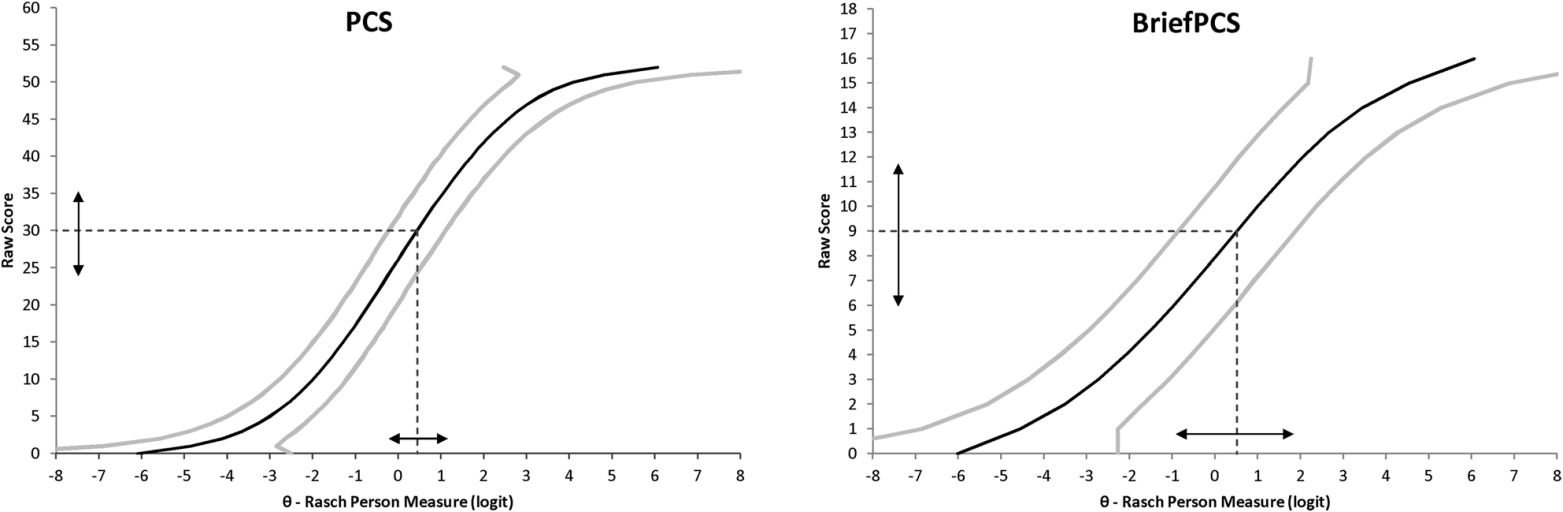
Conversion graph from total raw score (y-axis) to Rasch person measure (x-axis) for PCS and BriefPCS, with 95% confidence intervals (CI_95_, grey lines). Examples related to a score of 30 points for PCS (on the left) and of 9 points for BriefPCS (on the right): arrows delimit the CI_95_ for those values (see also text).

To exemplify the effect of the difference in measurement precision between the original scale and its short-forms, Fig. 3 reported the raw score to Rasch person measure conversion graph (with related CI_95_) for the PCS and BriefPCS, with an example based on the proposed cut-offs for clinically relevant level of catastrophizing. In the PCS, the cut-off score of 30 points corresponded to a Rasch measure of 0.40 logits (with SE 0.34), and accordingly the CI_95_ ranged from − 0.23 to 1.10 logits (~ 12 points of raw score). In BriefPCS, the cut-off score of 9 points corresponded to a Rasch measure of 0.51 logits (with SE 0.70) and the CI_95_ ranged from − 0.86 to 1.88 logits (~ 5.5 points of raw score). Thus, the relative CI_95_ (ratio between the CI_95_ and the maximum score range) of the PCS is ~ 23% of the score range (~ 12 points of CI_95_ divided by 52 points), while that of the BriefPCS is ~ 34% of the score range (5.5 points of CI_95_ divided by 16 points).

## Discussion

This is the first study to compare the psychometric characteristics of PCS and its short forms in individuals with chronic non-specific LBP, using Rasch analysis and focusing on their respective measurement precision in estimating the individual’s pain catastrophizing level.

We confirmed a correct functioning of the 5-option rating scale, common to the four questionnaires, in line with Nishigami et al.^13^. Our analysis of the internal construct validity of the four scales showed their substantial unidimensionality and a good fit of most items to the Rasch model (Table 1). This finding is reasonably consistent with previous Rasch studies^11–13^, although a detailed comparison cannot be performed due to differences in design and methodology (statistical models and software used; study protocol and methods; population, etc.). In particular, the most important misfit—that of item # 7 “I keep thinking of other painful events”—was already reported in two studies^12,13^: its responses showed a slightly higher variability than expected by the Rasch model, probably due to differences in the individuals’ history of painful events. Anyhow, according to Winsteps manual, this level of underfit (i.e. of unexpectedly high response variability), while unproductive for effective measurement, does not degrade it. As for the other cases of misfit, the underfit of item # 13 (“I wonder whether something serious may happen”) in the PCS-6 and of item # 4 (“It is awful and I feel that it overwhelms me”) in the BriefPCS was borderline and negligible. The overfit (i.e. a response pattern that is overly predictable) of items # 2 (“I feel I can’t go on”) and # 6 (“I become afraid that the pain will get worse”) in the PCS and of item # 10 in the BriefPCS does not deteriorate the measurement quality^24^. As a general rule, based on previously developed short scales, if an item provides acceptable clinical information, it should not be removed^29^.

In comparison with the PCS values, the three short forms showed lower person reliability and Cronbach’s alpha (as expected), and a higher number of minimum scores, particularly in the BriefPCS where the score distribution displayed a positive skewness (Table 2).

A comparison of the measurement precision of the four scales in estimating the individual’s level of pain-catastrophizing is shown in Figs. 1 and 2, examining both SEs and TIFs. This information given by a TIF is more precise and detailed that than coming from classical test theory statistics, where reliability is typically represented by a global value (e.g. Cronbach’s alpha) related to the whole score range, in spite of the known variability of error variance along the score continuum^29–31^. Higher TIF values correspond to lower SE values, and both indicate a higher conditional measurement precision. In any scale, the TIF should ideally be fairly high over an extended zone of the person ability range, with a relatively lower precision at the extremes (where the SE may also result inaccurate)^34^. However**,** in Fig. 2, only the TIF of the PCS exhibited an acceptable precision (SE ≤ 0.5) for a large range of θ (spanning about 6 logits), while the three PCS short versions displayed a modest (SE > 0.5) measurement precision over the whole scale range.

A high SE (here, standard error of the estimate of pain catastrophizing level) is undesired because it indicates inaccurate estimates of a person’s true score, due to high variability of the observed scores around the true score (the so-called “propensity distribution”, the hypothetical distribution of test scores obtained in a large number of test repetitions). It means there is a higher risk of making erroneous clinical decisions at individual level, based on imprecise values^18^.

In the example reported in the Results section and Fig. 3, measurement precision of the PCS full scale was clearly higher than that of the BriefPCS (the relative CI_95_ of the latter was about 50% larger than that of the PCS). At the analyzed PCS cutoff score for clinically relevant levels of catastrophizing (30 points, corresponding to a person ability θ = 0.40 logits)^33^, the SE was acceptable (0.34) (Fig. 1) and the test information quite high (> 8) (Fig. 2). Nonetheless, the interval of uncertainty around the cutoff (i.e. the conditional CI_95_) ranged from ~ 24 to 36 points of raw score (-0.23 to 1.10 logits). This result has important implications for clinical decision making based on PCS scores, in terms of the scale’s diagnostic accuracy (classification of high vs. low pain catastrophizers)^17,19,35^. Thus, we recommend that the present PCS cutoff scores of 24^36,37^ or 30^33^ points for a clinically relevant level of catastrophizing thoughts should not be rigidly applied for classification and clinical decision making in individuals. Further research about them (and the error margins around the cutoff) is needed. We also recommend caution when interpreting the change induced by targeted programs aimed at reducing catastrophizing thoughts^37–39^.

Our results highlight that very short scales (less than 10 items) even if composed of the best available items—despite their advantages for group research—cannot be used as a substitute for longer scales in individual decision-making. This*,* due to their inherent lower precision^17–19^*,* that negatively influences the accuracy of clinical decisions, whether the target be classification of the individual (e.g. for diagnosis), change assessment, or other. Moreover, test shortening (using different strategies) may have produced subsets of items not fully covering the construct of interest, and thus additional research on their content validity is advisable. Lastly, needless to say the shortened versions do not allow to properly examine other aspects of pain catastrophizing, such as how each PCS subdomain contributes to change in the pain outcome^40,41^ or can be targeted by different interventions^39^.

Overall, this study confirms—in people with chronic non-specific LBP—the sound psychometric characteristics of the 13-item PCS, including its essential unidimensionality and acceptable measurement precision over a fairly large range of scores^9^. Conversely, the low measurement precision of the PCS shortened versions warns against using them for decision-making in individuals^14,42^, although additional research in the different patient populations is warranted^43^. Accordingly, any proponents of a PCS short-form^13–16,44^ need to better clarify the measurement purposes and strengths/limitations of their new scale (e.g. regarding screening, status or severity assessment, prognosis, classification etc.), including its ability to render valid group-level vs. individual-level statistics and diagnostic accuracy^19,33,35^.

### Limitations

Our study has some potential limitations. First, we used a sample of outpatients with chronic non-specific LBP drawn from four rehabilitation facilities*,* and therefore the results cannot be generalized to other contexts or chronic pain conditions. Second, scores for the PCS short forms were extracted from those of the full PCS; in theory, it is uncertain how respondents would have answered if they had completed a shortened version per se. However, it is unlikely that participants’ responses would substantially differ, considering the scale length of the original version (13 items) and its administration time (less than 5 min)^20^. We used the same mode of data extraction from the full PCS version as was used in all previous studies on PCS short-forms^13–16^. However, studies administering the actual short forms of PCS would be useful to corroborate the present findings.

## Conclusion

In conclusion, this is the first study to examine (through their test information function) the measurement precision of PCS and its three short-forms (PCS-6, PCS-4, and BriefPCS) in estimating individual pain catastrophizing. Where clinical decisions on single subjects are the target, the original 13-item PCS should be preferred to its shortened versions and—pending further research on this topic—the herein reported confidence intervals for PCS true scores (i.e. the indication of how much uncertainty there is in the estimate of this parameter) should be taken into account.

## Data Availability

The datasets generated and analyzed during the current study are available from the corresponding author on reasonable request.

## Abbreviations

PCS: Pain Catastrophizing Scale
LBP: Low back pain
TIF: Test information function
PCS-6: 6-Item Pain Catastrophizing Scale
PCS-4: 4-Item Pain Catastrophizing Scale
BriefPCS: Brief Pain Catastrophizing Scale
MnSq: Mean-square statistics
PCAR: Principal component analysis of standardized residuals
